# Asiaticoside might attenuate bleomycin‐induced pulmonary fibrosis by activating cAMP and Rap1 signalling pathway assisted by A2AR

**DOI:** 10.1111/jcmm.15505

**Published:** 2020-06-16

**Authors:** Jing Luo, Ting Zhang, Chengwei Zhu, Junwei Sun, Wenjing Zhu, Wenxiu Ai, Xiaoying Huang, Xiaobing Wang

**Affiliations:** ^1^ Department of Rheumatology The First Affiliated Hospital of Wenzhou Medical University Wenzhou China; ^2^ Department of Pulmonary and Critical Care Medicine The First People's Hospital of Wenling Zhenjiang China; ^3^ Division of Pulmonary Medicine First Affiliated Hospital of Wenzhou Medical University Key Laboratory of Heart and Lung Wenzhou China

**Keywords:** adenosine A2A receptor, adenylate cyclase, asiaticoside, cAMP and Rap1 signalling pathway, pulmonary fibrosis

## Abstract

Asiaticoside (AS) has been reported to have protective effect on pulmonary fibrosis (PF). In this study, we aimed to explore the potential mechanism of the therapeutic role of AS and its relationship with A2AR in PF. Adenosine 2A receptor gene knockout (A2AR^−/−^) mice and wild‐type (WT) mice were used to establish bleomycin (BLM)‐induced PF models and were then treated with AS (50 mg/kg/d). Pulmonary inflammation and fibrosis were observed in the PF model with much higher severity in A2AR^−/−^mice than that in WT mice and AS significantly alleviated lung inflammation and fibrosis; however, it was less effective in A2AR^−/−^ mice than in WT mice via histopathological analysis. Using RNA sequencing analysis, we found up‐regulated differentially expressed genes (DEGs) in BLM group were enriched in immune and inflammation‐associated pathways compared with control group. There were 242 common DEGs between down‐regulated in BLM vs control group and up‐regulated in BLM + AS vs BLM group, which were enriched in cAMP and Rap1 signalling pathways. Furthermore, the expression of five key factors of these two pathways including adenylate cyclase (*ADCY1*, *ADCY5*, *ADCY8*, *cAMP* and *Rap1)* were confirmed up‐regulated by AS with the presence of A2AR. Therefore, AS might attenuate BLM‐induced PF by activating cAMP and Rap1 signalling pathways which is assisted by A2AR, making it a promising therapeutic optional for PF.

## 1 INTRODUCTION

Idiopathic pulmonary fibrosis (IPF) is a chronic, progressive interstitial lung disease characterized by the aberrant proliferation of fibroblasts and excessive extracellular matrix (ECM) with poor prognosis.^1^, ^2^ It is estimated that over five million people were affected by IPF globally with an average survival time of only 2‐3 years.^3^ Although the aetiology of IPF remains unknown, possible molecular mechanisms have been unravelled, encompassing overactivation and excess proliferation of myofibroblast, increased oxidative stress, alterations of growth factors expression, and genetic and epigenetic variations.^4^, ^5^, ^6^ Despite tremendous progress in understanding of the pathogenesis of IPF, the therapeutic choice of this disease is limited. The newly developed anti‐fibrotic drugs, nintedanib and pirfenidone, showed preferable inhibition of the progression of the fibrotic process in IPF; however, their usage is restricted due to their high price and side effects.^7^, ^8^ Therefore, there is still an unmet need for effective treatments with good safety.

Adenosine is an endogenous purine nucleoside that regulates various physiological effects by binding to high‐affinity adenosine receptors.^9^, ^10^ Adenosine 2A receptor (A2AR), one of adenosine receptors, has been found associated with the formation of fibrosis in various organs. A2AR was considered as a pathogenic factor in liver and skin fibrosis, and the antiviral drug tenofovir could effectively prevent liver and skin fibrosis through down‐regulating the level of adenosine.^11^, ^12^ However, more studies demonstrated that A2AR played a protective role in pulmonary, cardiorenal and renal fibrosis through inhibiting inflammatory cytokine expression and regulating endothelial cell function.^13^, ^14^, ^15^ The knockout of A2AR would exacerbate pulmonary damage.^16^ Down‐regulated A2AR was detected in severe IPF patients by Zhou's study, and A2AR‐knockout mice were found more sensitive to BLM‐induced lung injury in Scheibner's study.^13^, ^17^ More importantly, CGS21680, the A2AR agonist, was found to effectively inhibit inflammation and fibrosis in the lung, and A2AR knockout (A2AR^−/−^) mice have been found to be more sensitive than A2AR^+/+^ mice to bleomycin (BLM)‐induced lung injury.^13^, ^16^ Those studies indicated that A2AR played a crucial role in inhibiting pulmonary fibrosis as an endogenous protective receptor.

Asiaticoside (AS) is a triterpenoid saponin purified from the plant Centella asiatica with various biological effects, such as antioxidant, anti‐inflammatory and anti‐hepatofibric effects.^18^, ^19^, ^20^ Centella asiatica is a traditional Chinese medicine, which is widely used to wound healing and alleviating the symptoms of interstitial lung disease.^21^, ^22^ Tang et al found AS could inhibit the proliferation of fibroblasts and the expression of type I and type III collagen protein in the process of wound healing.^23^ Moreover, our previous study showed that AS can inhibit the expression of IL‐4, TNF‐α, and TGF‐β1 and increase the expression of A2AR in the lungs thus preventing the development of BLM‐induced pulmonary fibrosis in rats.^24^ However, the exact mechanism for the amelioration of BLM‐induced pulmonary fibrosis after treatment with AS remains unclear and requires further investigation. In this study, we used BLM‐induced PF model in wild‐type and A2AR^−/−^ mice and treated them with AS respectively to investigate the effect of AS on PF and to further explore potential mechanism of its the therapeutic role and its relationship with A2AR.

## 2 MATERIALS AND METHODS

### 2.1 Mouse models establishment and treatments

Forty‐five male wild‐type (WT) BALB/c mice were purchased from Beijing Vital River Laboratory Animal Technology Company, and Forty‐five male A2AR^−/−^ mice on BALB/c background were purchased from the Jackson Laboratory. BLM hydrochloride was purchased from Nippon Kayaku Co. (Tokyo, Japan), and AS (Sigma, USA) was diluted with normal saline. All WT BALB/c mice and A2AR^−/−^ mice were bred in the Laboratory Animal Centre of Wenzhou Medical University. The 8‐ to 10‐week‐old male WT mice (n = 45) and A2AR^−/−^ mice (n = 45) with weighing 20‐25 g were chosen for subsequent pulmonary fibrosis (PF) model establishment. These mice were housed in a specific pathogen‐free room with free access to water and food and underwent a 12‐hour light‐dark cycle with a controlled temperature (23 ± 2°C) and humidity (60 ± 10%). All experimental protocols were approved by the Animal Ethical Committee of Wenzhou Medical University.

The 45 WT mice and 45 A2AR^−/−^ mice were randomly divided into three groups respectively (15 mice each): a WT normal control (control) group, a WT BLM model (BLM) group, a WT BLM‐ and AS‐treated (BLM + AS) group, an A2AR^−/−^ normal control (KO) group, an A2AR^−/−^ BLM model (KOB) group, and an A2AR^−/−^ BLM‐ and AS‐treated (KOAS) group. Mice were anaesthetized by intraperitoneal injection with 20% urethane(1 mL/100 g), and BLM (5 U/kg) dissolved in sterile saline was intratracheally injected into the mice of the BLM, BLM + AS, KOB and KOAS group for inducing pulmonary fibrosis. Meanwhile, the mice in the control and KO group were given the same volume of sterile saline instead of BLM. From day 1 after intratracheal injection, the BLM + AS and KOAS group were given AS at a dose of 50 mg/kg/day by gastric perfusion and other group received sterile saline as control. Subsequently, the mice were euthanized by cervical dislocation on the day 28 and lung tissues were harvested for subsequent experiments.

### 2.2 Pathological assessment of inflammation and fibrosis in lung tissues with haematoxylin‐eosin and Masson's trichrome staining

The paraffin‐embedded blocks of lung tissues (1 mm^3^) were cut into 4 μm‐thick sections and stained with haematoxylin‐eosin (HE) and Masson's trichrome for pathological assessment. Four fields per section were randomly chosen under a magnification of 400×. To evaluate the severity degree of pulmonary interstitial tissue, the inflammation scores were graded based on the following criteria based on the results of HE staining: 0 (none), no alveolitis; 1+ (mild), thickening of the alveolar septum by mononuclear cells infiltration with limiting focal involvement, pleural lesions occupying less than 20% in the lung and with complete preservation of the alveolar architecture; 2+ (moderate), an extensive alveolitis involving 20%‐50% in the lung and still predominantly pleural based; 3+ (severe), a diffuse alveolitis occupying more than 50% in the lung, with few consolidation of air spaces by the intra‐alveolar mononuclear cells and some intra‐interstitial and/or intra‐alveolar haemorrhagic areas. Moreover, the fibrosis scores were also graded based on the following criteria based on the results of Masson's trichrome staining: 0 (none), no evidence of fibrosis; 1+ (mild), focal regions of fibrosis occupied less than 20% in the lung and only involved the pleura and the interstitium of the subpleural parenchyma with mild distortion of alveolar architecture; 2+ (moderate), fibrosis involving 20%‐50% in the lung and the fibrosis area extends inward from the pleura but is still focal; 3+ (severe), widespread fibrosis occupying more than 50% in the lung, with extensive derangement of parenchymal architecture, including cystic air spaces lined by cuboidal epithelium.^25^

### 2.3 RNA extraction, cDNA library preparation, sequencing and data preprocessing

Total RNA from lung tissue of 5 mice in each group was isolated using TRIzol® Reagent (Invitrogen), and RNA purity was checked using the Nano Photometer® spectrophotometer (IMPLEN, CA, USA). The high quality of RNA (RNA integrity numbers (RIN) >9) for cDNA library preparations was assessed by Bioanalyzer 2100 system using the Agilent RNA 6000 Nano kit (Agilent Technologies, CA, USA). Sequencing libraries were constructed using NEBNext UltraTM RNA Library Prep Kit for Illumina (NEB, USA) with the input material of 3 μg of RNA per sample and subsequently, the prepared libraries were sequenced on an Illumina HiSeq 2000 platform. Then, raw reads were trimmed using Cutadapt adapters and low‐quality reads were filtered using Trim Galore.^26^ Quality control reports of sequence reads were obtained through FastQC software (http://www.bioinformatics.babraham.ac.uk/projects/fastqc/).^27^ Finally, the sequencing data were aligned to the mouse reference genome (mm10).^28^ The read counts files were filtered with low expression and normalized by the ‘DEseq2’ package.^22^

### 2.4 Identification of differentially expressed genes

DESeq and vst (variance stabilized transformation) function in ‘DEseq2’ package were used to normalize and transform the counts file. Subsequently, regularized log transformation was conducted to exhibit the expression of samples in violin plots by the R package ‘ggplot2’ and cluster of samples was shown in heatmaps by the ‘pheatmap’ package. Then, we performed a two‐dimensional principle component analysis (PCA) and hierarchical clustering to visualize the similarities and differences among different groups. The differentially expressed genes (DEGs) were identified based on the following criteria: adjust *P* value < .05 and the absolute value of log2 FC (fold change) > 1. All the DEGs were visualized in volcano plots using the ‘ggplot2’ package, and clustering heatmap of DEGs was drawn using the ‘pheatmap’ package. Ensembl Gene IDs of DEGs were converted into gene symbols IDs by using ‘org.Mm.eg.db’ and ‘clusterProfiler’ R package.^29^, ^30^

### 2.5 Functional analysis for DEGs and protein‐protein interactions network analysis

For function enrichment analyses, Gene Ontology (GO) enrichment and Kyoto Encyclopedia of Genes and Genomes (KEGG) pathway analyses were conducted by using ‘clusterProfiler’ R package with the ‘enrichGO’ and ‘enrichKEGG’ function. The top 10 GO terms and all KEGG terms with adjusted *P* < .05 were visualized graphically by R package ‘GOplot’.^31^ To further investigate the potential role of AS in attenuation of PF, the common DEGs between down‐regulated genes in BLM vs control group and up‐regulated genes in AS vs BLM group were chosen to construct the protein‐protein interactions (PPI) networks. The circos plots of common DEGs and heatmap of common KEGG pathways were drawn on Metascape tool (http://metascape.org) [^32^]. The PPI data of the common DEGs were downloaded from The Search Tool for the Retrieval of Interacting Genes (STRING) database ^33^ and PPI networks were structured and visualized by Cytoscape software. The Molecular Complex Detection (MCODE) plugin in Cytoscape was used to screen significant modules of the PPI network with MCODE scores > 5 as the cut‐off criteria.^34^

### 2.6 Validation of key genes through reverse transcription quantitative polymerase chain reaction (RT‐qPCR) and ELISA

Total RNA was isolated from lung tissue samples using TRIzol® reagent (Invitrogen) according to the manufacturer's protocol, and one microgram of the total RNA was used for the reverse transcription and qPCR using the GoTaq R 2‐Step RT‐qPCR System (Promega). 5 key genes (*ADCY1*, *ADCY5*, *ADCY8*, *cAMP* and *Rap1*) of each group were respectively assayed by qPCR on an Applied Biosystems Real‐Time PCR Instrument (ABI) with three steps. For each PCR detection, after enzyme activation at 95°C for 2 minutes, amplification of 95°C was performed for 40 cycles and was completed after 60°C for 60 seconds. For each example, the PCR was repeated at three times and the gene expression level of genes was measured according to comparative ∆Ct (∆∆Ct) method.

Part of the lung tissue of each mouse was homogenized in phosphate buffer saline (PBS) using the Polytron homogenizer (PT1200‐E, Kinematica AG), and the supernatants were gained after centrifugation at 1129 xg for 20 minutes using a centrifuge 5810 ® (Eppendorf, Germany). ELISA 96‐well plates kits of five proteins (*ADCY1*, *ADCY5*, *ADCY8*, *cAMP*, and *Rap1*) were used as the carrier with prepackaged enzyme‐labelled antibody. Subsequently, 150 μL stock solution was serially diluted into standard dilutions with different concentrations (120 ng/mL, 60 ng/mL, 30 ng/mL, 15 ng/mL, 7.5 ng/mL) to draw standard curves. 50 μL supernatants and 50 μL biotinylated antigen working solution were respectively added into each well and incubated at 37°C for 60 minutes. Following washing for five times, 50 μL avidin‐HRP was added into the wells and incubated at 37°C for 30 minutes again. After reduplicated washing, 50 μL of chromogenic reagent A and B were used to develop the stain for 10 minutes and 50 μL stop buffer was employed to stop the reaction. Finally, absorbance of each well was measured at 450 nm using a Varioskan Flash (Thermo Scientific, USA) and concentration of samples was calculated via ‘ELISAcalc’ software with logistic model based on the standard curve.

Finally, the relative expression of key genes and proteins was exhibited in box diagrams using ggboxplot function in the ‘ggpubr’ R package^35^ and the statistical differences were conducted by Wilcoxon test with *P* < .05. The schema chart for AS’s potential mechanism to attenuate PF was drawn through Adobe Illustrator CC 2019.

### 2.7 Statistical analysis

The inflammation scores and fibrotic scores were presented as $\bar{x}\pm s$ , and the comparison of between‐groups was performed using Wilcoxon test. *P* value less than .05 was considered as statistical significance.

## 3 RESULTS

### 3.1 AS alleviated pulmonary inflammation of BLM‐induced pulmonary fibrosis

**Figure 1:**
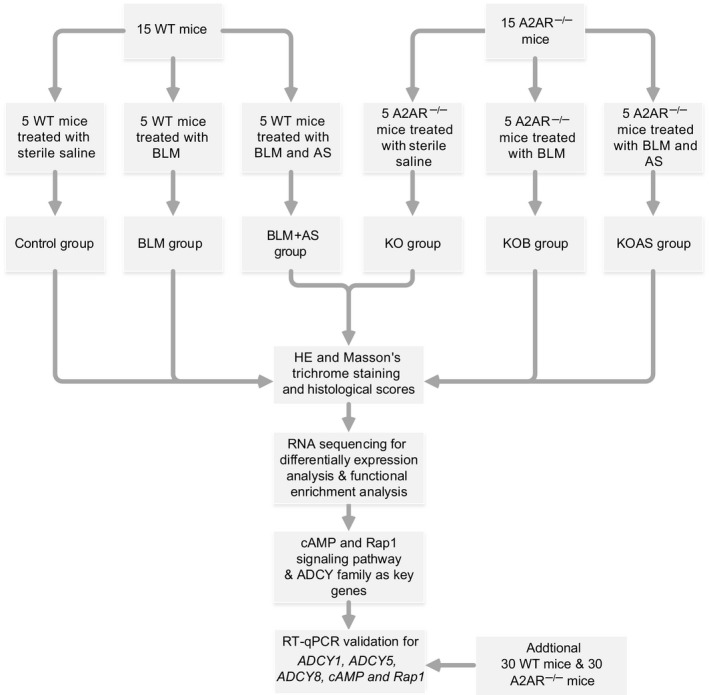
The summary and description of the study workflow. WT, wild‐type; A2AR, adenosine 2A receptor; BLM, bleomycin; AS, asiaticoside; HE, haematoxylin‐eosin

**Figure 2:**
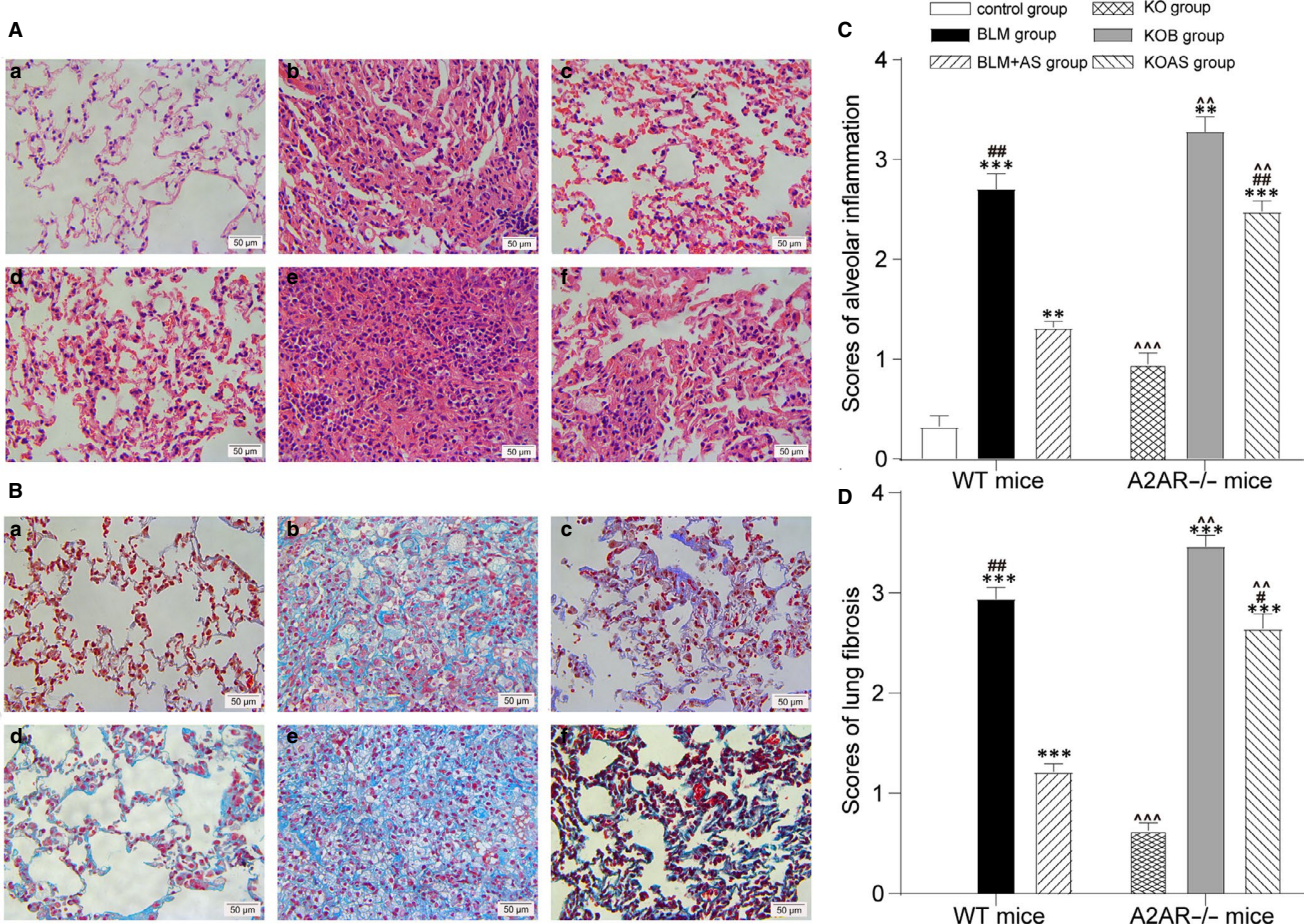
AS and A2AR alleviated pulmonary histopathological changes of BLM‐induced pulmonary fibrosis model. A, Images of HE staining of pulmonary tissue sections (light microscopy, 400×) in the control group (a); BLM group (b); BLM + AS group (c); KO group (d); KOB group (e) and KOAS group (f). The scale bars represent 50 μm. B, Images of Masson's trichrome staining of pulmonary tissue sections (light microscopy, 400×) in the control group (a); BLM group (b); BLM + AS group (c); KO group (d); KOB group (e) and KOAS group (f). The scale bars represent 50μm. C, Histogram showing scores of alveolar inflammation in different group $\bar{x}\pm s$ ; n = 5 in each group; D. Histogram showing scores of lung fibrosis in different group; $\bar{x}\pm s$ ; n = 5 in each group; *P < .05, **P < .01; ***P < .005, ‘*’ means comparison of BLM vs control, BLM + AS vs control, KOB vs KO and KOAS vs KO group; #*P* < .05, ##*P* < .01; ###*P* < .005, ‘#’ means comparison of BLM + AS vs BLM and KOAS vs KOB group; ^*P* < .05, ^^*P* < .01; ^^^*P* < .005, ‘^’ means comparison of KO vs control, KOB vs BLM and KOAS vs BLM + AS group

Figure 1 showed the workflow of our study. The pulmonary fibrosis models were successfully established after BLM treatment. HE staining revealed that no obvious pathological changes were observed in control group while mild inflammatory cells infiltration observed in KO group (Figure 2A a&d). In PF model group (BLM and KOB group), the obvious destruction of alveolar structure, thickening of alveolar walls and a large amount of collagen depositing were found. Moreover, the inflammation and fibrosis were much more severe in KOB group than that in BLM group (Figure 2A b&e). However, pulmonary inflammation, alveolar structural damage and collagen deposition were much alleviated in BLM + AS group. Of note, KOAS group show less improvement than that in BLM + AS group (Figure 2A c&f). Consistent with HE staining, Masson's trichrome staining showed that no obvious pathological change in control group while a small amount of collagen was deposited in the pulmonary interstitium in KO group (Figure 2B a&d). In contrast, BLM induced large amounts of collagen deposition in BLM and KOB groups, and the deposition was even more severe in A2AR^−/−^ mice than in WT mice (Figure 2B b&e). Moreover, BLM + AS group showed alleviated collagen deposition, more obvious in WT mice was better than in A2AR^−/−^ mice (Figure 2B c&f). In addition, the scores of alveolar inflammations and lung fibrosis were much higher in model groups (BLM and KOB group) than control groups (control and KO group) and significantly reduced alveolar inflammation scores were detected in treatment groups (BLM + AS and KOAS group) (Figure 2C and D) (*P* < .01). The A2AR^−/−^ mice group (KO, KOB and KOAS group) displayed a higher level of alveolar inflammation and pulmonary fibrosis than WT mice groups (control, BLM and BLM + AS group) (*P* < .01, *P* < .005).

### 3.2 Activated immune response participated in BLM‐induced pulmonary fibrosis

**Figure 3:**
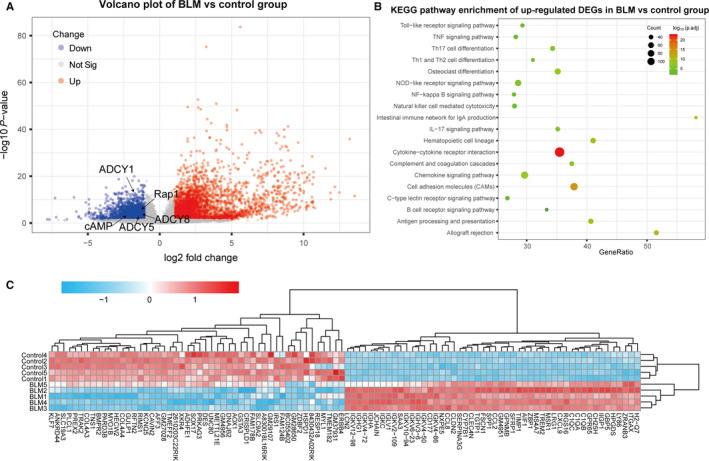
Results of differential expression analysis between BLM and control group. A, Volcano plot showing DEGs in BLM vs control group; (B) KEGG analysis showing up‐regulated DEGs of BLM vs control group were major enriched in immune‐related pathways; (C) The heatmap of top 100 DEGs (50 up‐ and 50 down‐regulated DEGs) showed significant different clustering between BLM and control group

**Figure 4:**
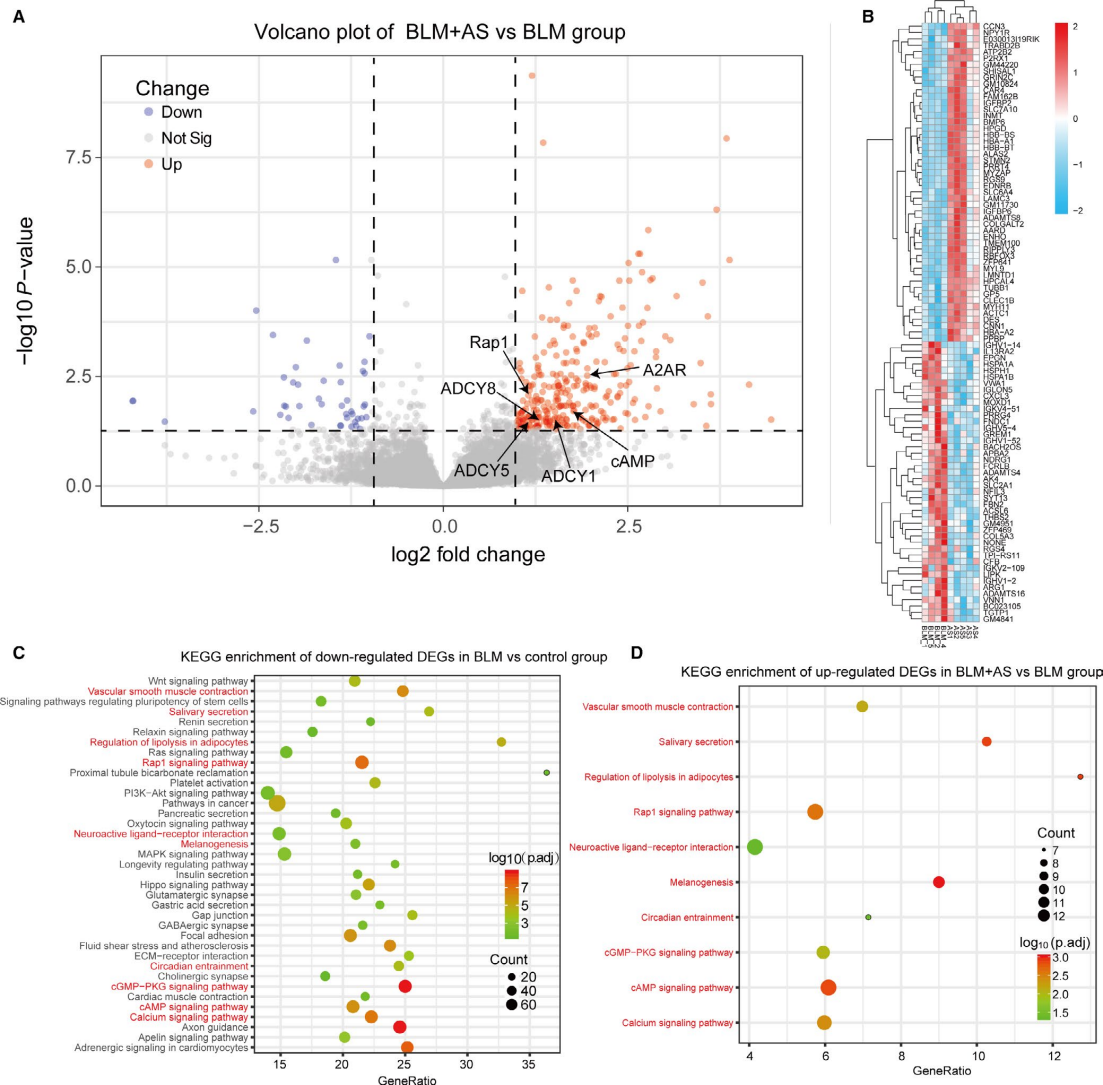
Results of differential expression analysis between BLM + AS and BLM group. A, Volcano plot showing DEGs in BLM + AS vs BLM group; (B) The heatmap of top 100 DEGs (50 up‐ and 50 down‐regulated DEGs) showed significant different clustering between BLM + AS and BLM group. C and D, Bubble plots showing a high similarity of pathway analysis between down‐regulated DEGs in BLM group and up‐regulated DEGs in BLM + AS group, especially cAMP signalling pathway, Rap1 signalling pathway, cGMP‐PKG signalling pathway and calcium signalling pathway with significant difference

To better evaluate the gene expression features of BLM‐induced pulmonary fibrosis, we performed differential expression analysis between BLM and control group. The gene expression distribution of each sample was homogeneous and comparable after quality control. BLM and control group were generally separated into two distinct clusters by clustering and PCA analysis (Figure S1A). Using the cut‐off criteria (adjust *P* value < .05 and |log FC| > 1), we identified a total of 5323 DEGs including 3236 up‐regulated genes and 2087 down‐regulated genes and the heatmap of top 100 DEGs (50 up‐ and 50 down‐regulated DEGs) showed significant different clustering between two group (Figure 3A and C). As shown in Figures S1B and S3B, GO analysis indicated up‐regulated DEGs were mainly enriched in biological processes of positive regulation of cytokine production and T‐cell activation, whilst KEGG analysis indicated up‐regulated DEGs were associated with inflammatory and immune‐related pathways such as cytokine‐cytokine receptor interaction and cell adhesion molecules (CAMs). In addition, the down‐regulated DEGs were associated with biological process of signal regulation including cAMP signal pathway, Rap1 signal pathway, cGMP‐PKG signalling pathway and calcium signalling pathway (Figure 4C, Figure S1C).

### 3.3 AS might inhibit BLM‐induced pulmonary fibrosis through activating cAMP and Rap1 signalling pathway

To further investigate the potential role of AS in the treatment of BLM‐induced pulmonary fibrosis, we compared the differential expression manifestation between BLM + AS group and BLM group. After eligible quality control, 342 DEGs including 298 up‐regulated genes and 44 down‐regulated genes were identified as showed in the volcano plot (Figure 4A) with significant discriminatory capacity in the heatmap of top 100 DEGs (Figure 4B). Interestingly, the up‐regulated DEGs in AS were enriched in those signal regulation pathways which down‐regulated in BLM group encompassing cAMP signal pathway Rap1 signal pathway, cGMP‐PKG signalling pathway and calcium signalling pathway (Figure 4C and D). There was no significantly enriched pathway in the down‐regulated DEGs of BLM + AS group.

### 3.4 Identification of key genes regulated by the treatment of AS

**Figure 5:**
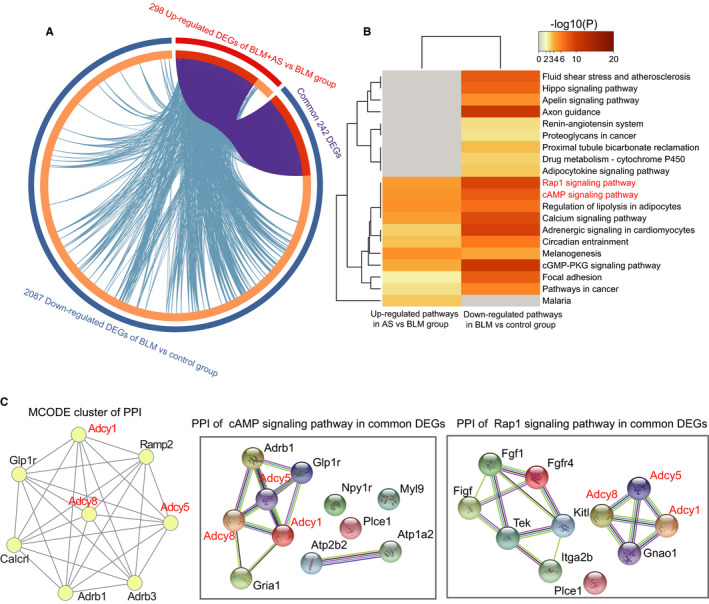
Identification of key pathways and genes for the treatment of AS. A, Circle plots showing 242 common DEGs between down‐regulated DEGs in BLM and up‐regulated DEGs in BLM + AS group. B, Pathway analysis identifying cAMP signalling pathway and Rap1 signalling pathway with significant *p* value. C, The core module (with the MCODE score of 8) from the PPI network of common DEGs showing ADCY1, ADCY5, ADCY8 played essential role in regulating the network. D and E, The PPI of cAMP and Rap1 signalling pathway using associated genes in common DEGs

Further, 242 common DEGs between down‐regulated DEGs in BLM group and up‐regulated DEGs in BLM + AS group were screened (Figure 5A). These DEGs were enriched in the cAMP, Rap1, cGMP‐PKG and calcium signal pathway with significant *p* value using Metascape tool and filtered into the PPI network, forming 218 nodes and 259 edges with average node degree of 2.38 (Figure 5B, Figure S3). Moreover, the most significant module (MCODE score = 8) was screened to identify key genes, including 8 genes (*ADCY1*, *ADCY5*, *ADCY8*, *GLP1R*, *CALCRL*, *ADRB1*, *ADRB3*, *RAMP2*) which participate in the cAMP and Rap1 signal pathway (Figure 5C). Notably, the *ADCY* family (*ADCY1*, *ADCY5*, *ADCY8*) were found at central position in the network as the common key genes between cAMP and Rap1 signalling pathways. The protein‐protein interactions of cAMP and Rap1 signal pathway were displayed in Figure 5D. Based on above findings, we further identified key genes (*ADCY1*, *ADCY5*, *ADCY8*) in cAMP signal pathway and Rap1 signal pathway which were potentially regulated by the treatment of AS in BLM‐induced pulmonary fibrosis.

### 3.5 Role of A2AR in BLM‐induced pulmonary fibrosis

**Figure 6:**
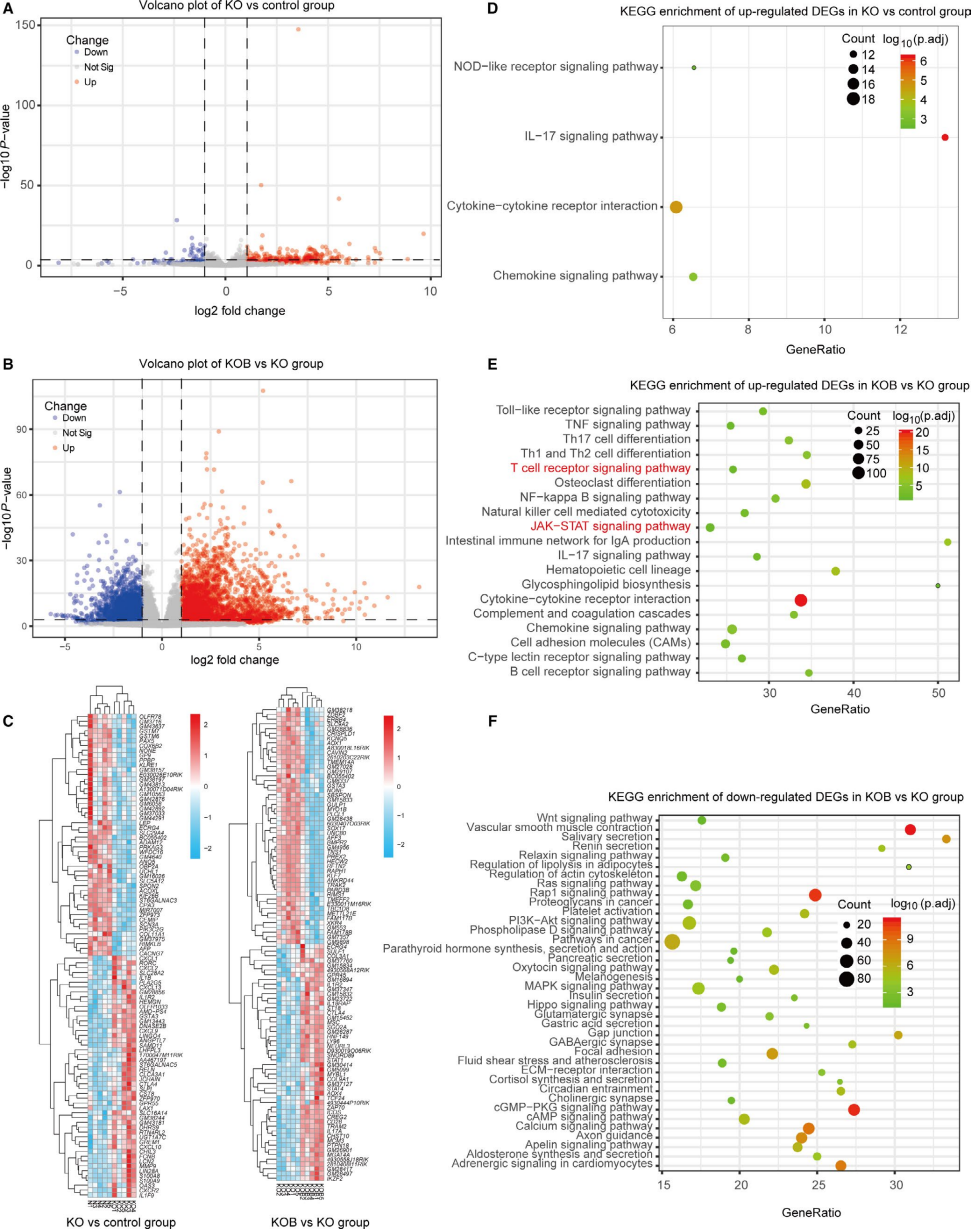
Results of differential expression analysis of KO vs control and KOB vs KO group. A and B, Volcano plot showing DEGs of KO group and KOB group. C, The heatmap of top 100 DEGs (50 up‐ and 50 down‐regulated DEGs) showed significant different clustering in KO vs control and KOB vs KO. D, KEGG analysis showing up‐regulated DEGs of KO group were major enriched in inflammatory pathways including IL‐17 signalling pathway, cytokine‐cytokine receptor interaction, chemokine signalling pathway and NOD‐like receptor signalling pathway. E, Bubble plots showing a high similarity of pathway analysis between up‐regulated DEGs in BLM group and up‐regulated DEGs in KOB group while JAK‐STAT signalling pathway and T‐cell receptor signalling pathway are equal in KOB group. F, Pathway analysis of down‐regulated DEGs in KOB group indicating cAMP signalling pathway, Rap1 signalling pathway and cGMP‐PKG signalling pathway with significant difference

A2AR has been considered as protective factors in pulmonary fibrosis and our previous studies found AS could up‐regulate the expression A2AR to inhibit inflammatory process in pulmonary diseases. In this study, bleomycin did not alter the expression of A2AR while AS can significantly elevate the expression of A2AR in WT mice (with logFC = 1.72, adjust *P* value = .004; Figure 4A). To investigate the mechanism of its protective effect on pulmonary fibrosis, we also conducted differential expression analysis between KO and control group. A total of 357 up‐regulated and 94 down‐regulated DEGs were identified after quality control (Figure 6A, Figure S4A). The functional enrichment analysis of these up‐regulated DEGs indicated cytokine‐ and chemokine‐related pathways were significantly activated after A2AR knockout, including IL‐17 signalling pathway, cytokine‐cytokine receptor interaction, chemokine signalling pathway and NOD‐like receptor signalling pathway (Figure 6D). No significant enrichment pathway was observed in the down‐regulated DEGs of KO group when compared to control group.

The comparison of KOB vs KO group and KOAS vs KOB group was also performed respectively. 3328 up‐regulated and 2158 down‐regulated DEGs were identified with significant discriminatory capacity between KOB and KO group (Figure 6B and C) and the pathway enrichment of KOB maintained a high similarity to that of BLM (Figure 6E and F). However, there were only two up‐regulated DEGs (with the cut‐off: |logFC| >1 and adjust *P* value < .05) and no significantly enriched pathway was found between KOAS and KOB group (Table S1). However, 626 other genes were also up‐regulated and 773 other genes down‐regulated (with |logFC|>0, *P* value < .05), however not reach to the strict cut‐off criteria. Some of which involves in TGF‐β, ECM‐receptor, focal adhesion and hippo signalling pathways. Interestingly, the cAMP signalling pathway and Rap1 signalling pathway were also identified in the down‐regulated pathways of KOB, and two pathways (JAK‐STAT signalling pathway and T‐cell receptor signalling pathway) were found distinctive in the KOB group, compared with up‐regulated pathways in BLM group (Figure 6E). In addition, we also performed the comparison of KOB vs BLM group and KOAS vs BLM + AS group; however, there were a few DEGs and no significantly enriched pathway (Tables S2 and S3). Notably, three hub genes (*ADCY1*, *cAMP* and *Rap1*) were significantly down‐regulated in KOAS versus BLM + AS group (Table S2).

### 3.6 Validation of key genes in cAMP and Rap1 signal pathways regulated by AS in pulmonary fibrosis

**Figure 7:**
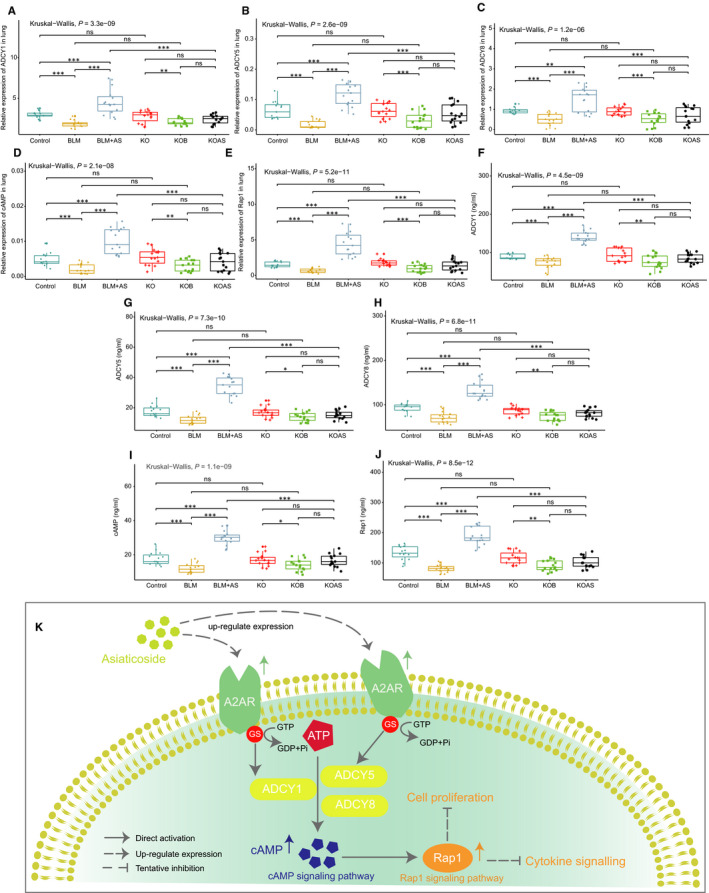
Results of RT‐qPCR and ELISA validation for cAMP and Rap1 signal pathway. A‐E, The genic expression of *ADCY1*, *ADCY5*, *ADCY8*, *cAMP* and *Rap1* was validated via RT‐qPCR in control, BLM, BLM + AS, KO, KOB and KOAS group. ***, *P* < .005; **, *P* < .01; *, *P* < .05; ns, no significant difference. F‐J, The protein expression of *ADCY1*, *ADCY5*, *ADCY8*, *cAMP* and *Rap1* was validated via ELISA in control, BLM, BLM + AS, KO, KOB and KOAS group. ***, *P* < .005; **, *P* < .01; *, *P* < .05; ns, no significant difference. K, The schema chart showing the hypothesis that AS may attenuate bleomycin‐induced pulmonary fibrosis by activating cAMP and Rap1 signalling pathway assisted by A2AR

To validate the effect of AS on the expression of the five key genes (*ADCY1*, *ADCY5*, *ADCY8*, *cAMP* and *Rap1*) in cAMP and Rap1 signal pathways in pulmonary fibrosis, we measured their expression at gene level and protein level by RT‐qPCR and ELISA experiments, respectively. It turned out the gene expression of *ADCY1*, *ADCY5*, *ADCY8*, *cAMP* and *Rap1* was dramatically decreased in BLM group and increased after the treatment of AS in WT mice. The gene expression of these hub genes also significantly decreased in KOB group while AS failed to up‐regulate the expression after the knockout of A2AR. However, those genes were remarkably activated by AS in BLM mice while there was no significant difference in KOAS group, which is consistent with the results of RNA sequencing (Figure 7A‐E). Furthermore, the ELISA revealed the expression of those genes at protein level was dramatically decreased in BLM group but increased after treated with AS in WT mice (BLM + AS group vs BLM group). Contrarily, the expression was also significantly reduced in KOB group and shows no elevated level in KOAS group. Moreover, it was also found those genes were significantly activated by AS assisted with A2AR (KOAS vs BLM + AS group) and there was no significant difference in KO vs control group and KOB vs BLM group, consistent with the results of RT‐qPCR (Figure 7F‐J). Finally, we draw a schema chart of the potential mechanism of the therapeutic role of AS in BLM‐induced pulmonary fibrosis, which was displayed in Figure 7K.

## 4 DISCUSSION

IPF is a chronic, progressive, lethal interstitial lung disorder. BLM‐induced pulmonary fibrosis in mice or rats model is most widely used, characterized by remarkable intestinal inflammation, excessive collagen deposition and lung tissue destruction.^36^ Although the cause of IPF is complex, epithelial to mesenchymal transition (EMT), fibroblast proliferation and differentiation, and persistent chronic inflammation were considered as some of the major significant mechanisms of IPF.^2^, ^37^, ^38^ Regarding to the role of inflammation in the process of pulmonary fibrosis, there are still some controversies over this subject. Mauviel et al and Chizzolini et al found TNF‐α could inhibit the synthesis of type I collagen and exert the inhibitory effects of CD4 + T cells on collagen production in dermal fibroblasts.^39^, ^40^ However, evidences based on animal model and human studies suggested that inflammatory responses induced by multiple immune cells could regulate existing fibrotic responses.^41^, ^42^, ^43^ Moreover, studies also demonstrated substantial inflammatory infiltration and collagen deposition were significant pulmonary features in BLM‐induced pulmonary fibrosis A2AR^−/−^ mice models, which was used in our study.^44^, ^45^ In accordance with previous research, we also found significant inflammatory infiltration and large amounts of collagen fibres deposition in BLM group and even more serious in KOB group, which indicated the PF model was established successfully by BLM and PF were even worsen in A2AR^−/−^ mice. It was noteworthy that several associated genes (such as TGF‐β, MMP9 and Wnt6) were up‐regulated in pulmonary fibrosis model, which coincided with previous studies.^46^, ^47^, ^48^

It has already been demonstrated that many signalling pathways participated in the progression of IPF, such as VEGF, PI3K‐Akt, TGF‐β and Wnt/β‐catenin signalling pathway.^49^ In addition, Garcia et al found several canonical pathways related to inflammatory and immune responses were involved in bleomycin‐induced PF, such as triggering receptor expressed on myeloid cells (TREM1) signalling, neuroinflammation signalling pathway, production of nitric oxide and reactive oxygen species.^50^ In our study, the functional enrichment analysis demonstrated immune‐related and inflammatory pathways were activated in BLM‐induced pulmonary fibrosis including innate immunity pathways (such as natural killer cell‐mediated cytotoxicity, toll‐like receptor signalling pathway and nod‐like receptor signalling pathway) and adaptive immune responses (such as B‐cell receptor signalling pathway, Th1‐ and Th2‐cell differentiation and Th17‐cell differentiation). Moreover, cytokine‐associated pathways (such as chemokine signalling pathway, CAMs, cytokine‐cytokine receptor interaction and NF‐kappa B signalling pathway) were also up‐regulated in BLM group indicating the activation of immune reaction and inflammation response in the progression of PF. Therefore, both inflammation and immunity involved in the BLM‐induced pulmonary fibrosis.

Although N‐acetylcysteine, glucocorticoids, pirfenidone and nintedanib have been approved for clinical use, there is still unmet need for optimal therapeutic choice for IPF.^51^ As an active monomer purified from Centella asiatica, AS belongs to the family of α‐aromatic alcoholic triterpenoid saponins with diverse pharmacological and biological activities. Besides, AS has already got approval to put into oral pills in China with good response and safety profile, which used for several indications, such as scleroderma and skin scar.^52^, ^53^, ^54^ In our study, oral administration method was also used with reference to previous studies and also based on our preliminary study which has test out the optimal therapeutic dose of AS in mouse model.^24^ Further, Weng et al revealed AS cannot be metabolized by cytochrome P450 in liver, but mostly be hydrolysed by intestinal bacteria.^55^ Our previous study had demonstrated that AS could inhibit the progression of hypoxia‐induced pulmonary hypertension through TGF‐β1/Smad signalling.^56^ In addition, madecassoside, another component of Centella asiatica, was reported to alleviate BLM‐induced pulmonary fibrosis in Lu's study.^57^ In this study, AS revealed significant effect on pulmonary fibrosis by reducing pneumonia inflammatory infiltrations and collagen deposition; however, the therapeutic effects of AS were poor if A2AR was knocked out, suggesting A2AR might play an essential role in the therapeutic mechanism of AS.

Furthermore, we found the up‐regulated DEGs‐associated enriched pathways after AS treatment were those down‐regulated in BLM group, such as cAMP signalling pathway, Rap1 signalling pathway and cGMP‐PKG signalling pathway. It was interesting that cAMP could directly regulate the Rap1 signalling pathway through activating the mediators Epac1 and Epac2 protein, contributing to inhibiting fibrosis and inflammation in lung.^57^, ^58^, ^59^ Anggoro's study confirmed that cAMP/Epac1 pathway could effectively limit the inflammation and fibrosis in BLM‐induced pulmonary fibrosis models.^60^ Moreover, cAMP and Rap1 signalling pathways were further identified as hub pathways for AS treatment based on functional enrichment and PPI analysis of common DEGs. *ADCY1*, *ADCY5* and *ADCY8*, which are significant components in cAMP signalling pathway,^61^ was identified as key genes according to MCODE analysis. Hence, it was assumed that AS might regulate the cAMP and Rap1 signal pathway through ADCY family to attenuate BLM‐induced pulmonary fibrosis.

In this study, some inflammatory pathways were up‐regulated in KO group compared with control group including nod‐like receptor signalling pathway, cytokine‐cytokine receptor interaction, chemokine signalling pathway and IL‐17 signalling pathway, which explained the susceptibility to pulmonary fibrosis after A2AR knockout. Huang et al found that A2AR‐null mice developed more severe pulmonary fibrosis than wild‐type mice after treated with BLM,^45^ which is consistent with our findings. Notably, JAK‐STAT signalling pathway and T‐cell receptor signalling pathway were uniquely activated in KOB group compared with up‐regulated pathways of BLM group, interpreting the more serious pulmonary damage after A2AR knockout. Therefore, we inferred that JAK‐STAT and T‐cell receptor signalling pathways may be responsible for the vulnerability of PF after treated with BLM in A2AR^−/−^ mice.

Previous studies demonstrated that the binding of adenosine to A2AR can activate ADCY by stimulatory G‐proteins (Gs), which leading to the activation of cAMP signalling pathway.^62^, ^63^ This was further confirmed by our findings that the gene knockout of A2AR down‐regulated this key pathway. However, ADCY can be up‐regulated by AS, which may then activate the cAMP and Rap1 signalling pathway especially with the presence of A2AR in BLM‐induced pulmonary fibrosis. We have found AS might increase the expression of A2AR which helps prevent the development of BLM‐induced pulmonary fibrosis in rats in our another study.^24^ In this study, we demonstrated that BLM cannot alter the expression of A2AR while AS significantly up‐regulated the expression of A2AR, which accorded with our previous study. Furthermore, we found much severe alveolar inflammation in KOB group and poor curative effect by AS in KOAS group. All these observations demonstrated that A2AR might play an important protective role in inhibiting pulmonary fibrosis and AS has potential effect with the assistance of A2AR. Finally, the expressions of those hub genes were still dramatically decreased with the treatment of AS in A2AR^‐/‐^ mice (KOAS vs BLM + AS group), suggesting that A2AR, as involved in ADCYs‐cAMP‐Rap1 pathways, might play a potential role in the therapeutic effect of AS on bleomycin‐induced pulmonary fibrosis.

However, there are some limitations in our study. First, the comparatively small sample sizes of each group using for RNA‐seq may inducing potential selection bias, although we have tripled the sample size when validating the expression of ADCY family. Besides, we conceived that AS might up‐regulate the expression of ADCY family to activate the cAMP and Rap1 signalling pathway assisted by A2AR to attenuate BLM‐induced pulmonary fibrosis; however, further in vivo and in vitro experiments are needed to investigate the specific functions of ADCY family and their detailed mechanism in the treatment of AS in pulmonary fibrosis.

In conclusion, we confirmed the protective effect of AS on attenuating BLM‐induced pulmonary fibrosis, which was compromised when A2AR gene was knocked out in the animal model. AS may attenuate bleomycin‐induced pulmonary fibrosis by activating cAMP and Rap1 signalling pathway assisted by A2AR. This study attempted the treatment of PF with AS and dug its potential underlying mechanisms with pathway exploration in hope to discover a promising therapeutic optional for PF.

## CONFLICT OF INTEREST

All authors have declared that no conflict of interest exists.

## AUTHOR CONTRIBUTION

**Jing Luo:** Methodology (equal); Writing‐original draft (lead). **Ting Zhang:** Writing‐original draft (equal). **Chengwei Zhu:** Software (equal); Validation (equal). **Junwei Sun:** Visualization (equal). **Wenjing Zhu:** Writing‐review & editing (equal). **Wenxiu Ai:** Resources (equal); Supervision (equal). **Xiaoying Huang:** Data curation (equal); Formal analysis (equal). **Xiaobing Wang:** Data curation (equal); Funding acquisition (lead); Project administration (lead). 

## ETHICAL APPROVAL

The study was approved by the Animal Ethical Committee of Wenzhou Medical University. All procedures of our experiments were in the guide for the Care and Use of Laboratory Animals published by the US National Institute of Health.

## Supporting information

Fig S1

Fig S2

Fig S3

Fig S4

Table S1‐S3

## Data Availability

The pathological data, RNA sequencing and RT‐qPCR and ELISA data are available under reasonable request.
