# Supplementary material for: Asiaticoside might attenuate bleomycin‐induced pulmonary fibrosis by activating cAMP and Rap1 signalling pathway assisted by A2AR
Source: J Cell Mol Med. 2020 Jun 16;24(14):8248–61. doi: 10.1111/jcmm.15505 (PMC7348182; doi:10.1111/jcmm.15505)

Supplementary Figure 1

A. Quality control for BLM vs control group

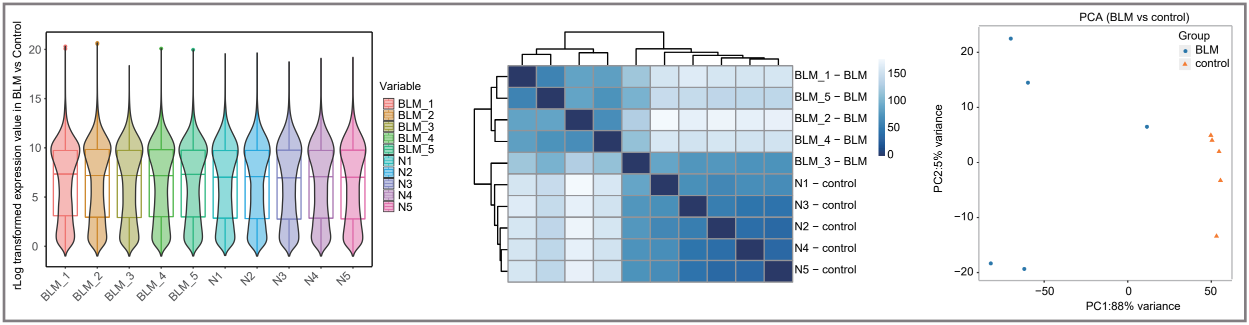

B. GO enrichment of up-regulated DEGs in BLM vs control group

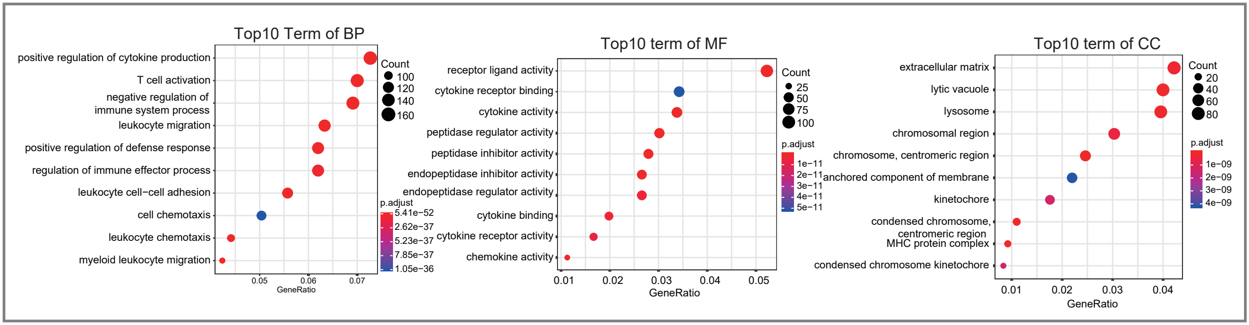

C. GO enrichment of down-regulated DEGs in BLM vs control group

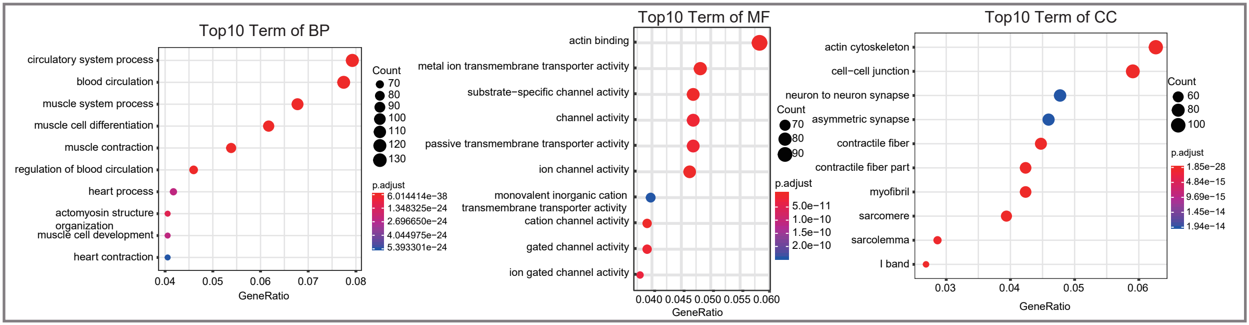

Supplement: Supplementary file 1 — Fig S1 [file JCMM-24-8248-s001.pdf]
