# Supplementary material for: Asiaticoside might attenuate bleomycin‐induced pulmonary fibrosis by activating cAMP and Rap1 signalling pathway assisted by A2AR
Source: J Cell Mol Med. 2020 Jun 16;24(14):8248–61. doi: 10.1111/jcmm.15505 (PMC7348182; doi:10.1111/jcmm.15505)

Supplementary Figure 2

A. Quality control for BLM+AS vs BLM group

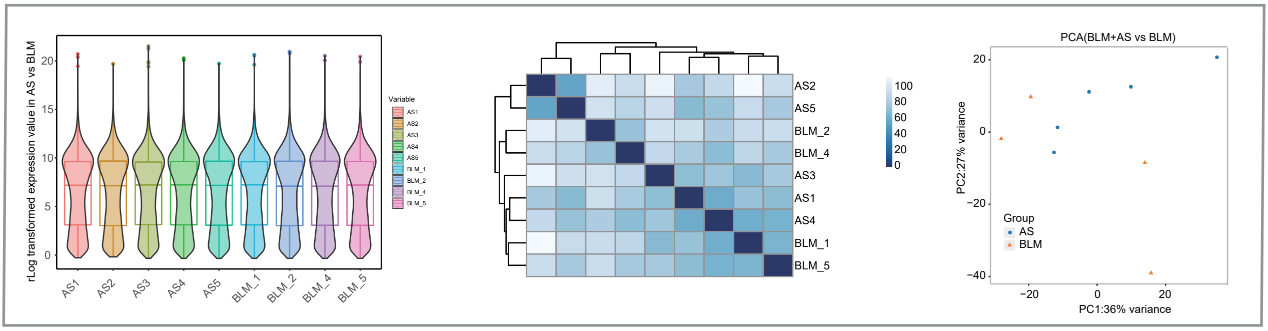

B. GO enrichment of up-regulated DEGs in BLM+AS vs BLM group

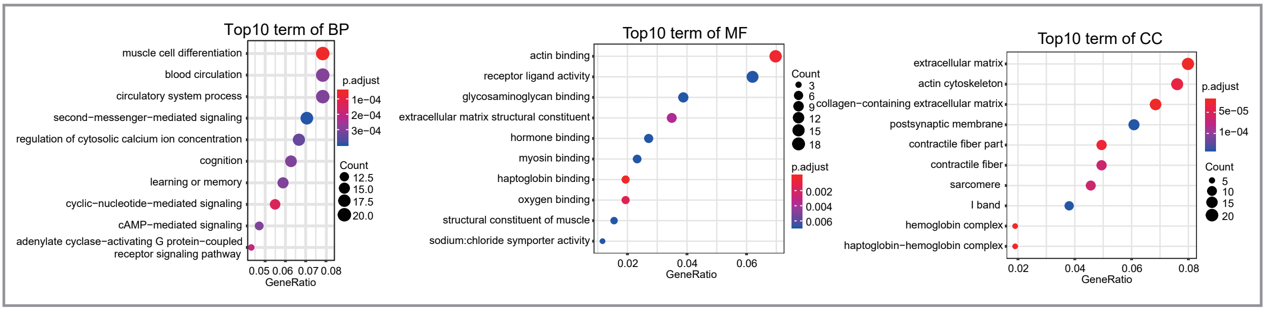

C. GO enrichment of up-regulated DEGs in KO vs control group

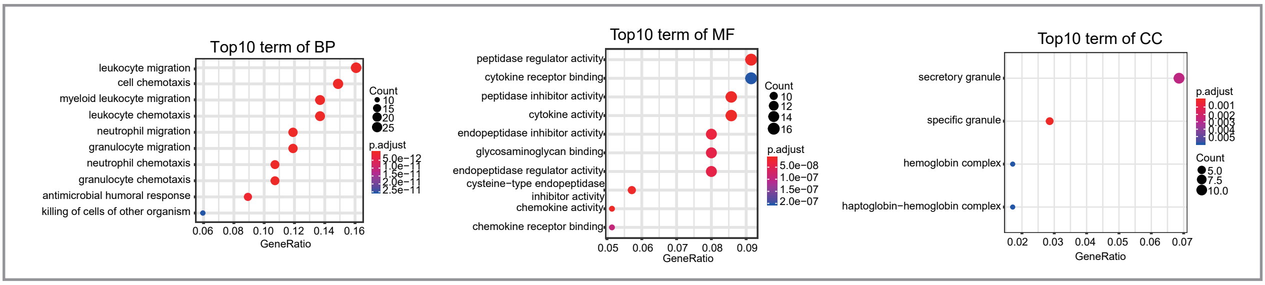

D. GO enrichment of up-regulated DEGs in KOB vs KO group

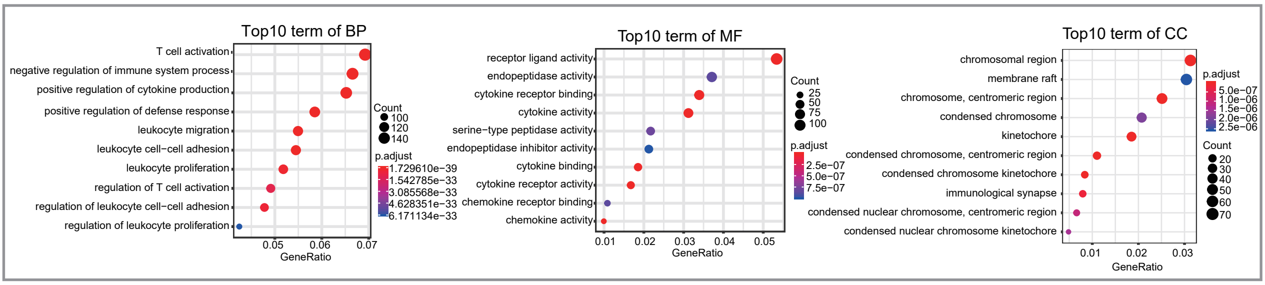

E. GO enrichment of down-regulated DEGs in KOB vs KO group

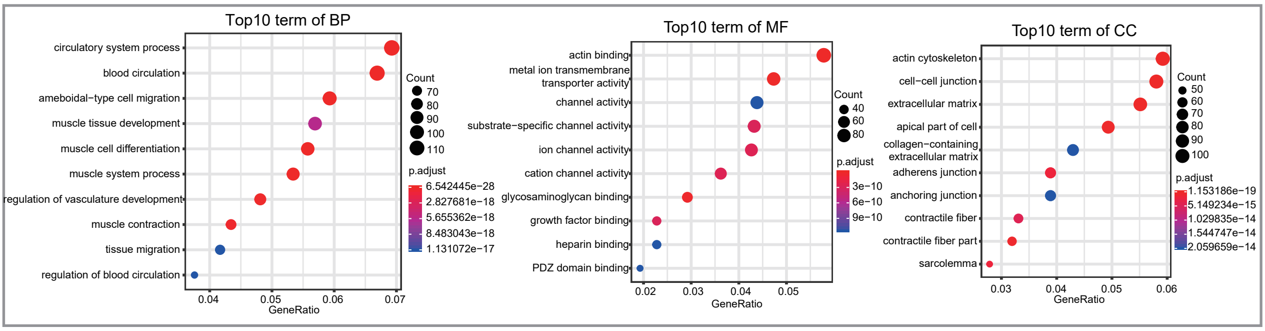

Supplement: Supplementary file 2 — Fig S2 [file JCMM-24-8248-s002.pdf]
