# Supplementary figures and images for: Asiaticoside might attenuate bleomycin‐induced pulmonary fibrosis by activating cAMP and Rap1 signalling pathway assisted by A2AR
Source: J Cell Mol Med. 2020 Jun 16;24(14):8248–61. doi: 10.1111/jcmm.15505 (PMC7348182; doi:10.1111/jcmm.15505)

Supplementary Figure3

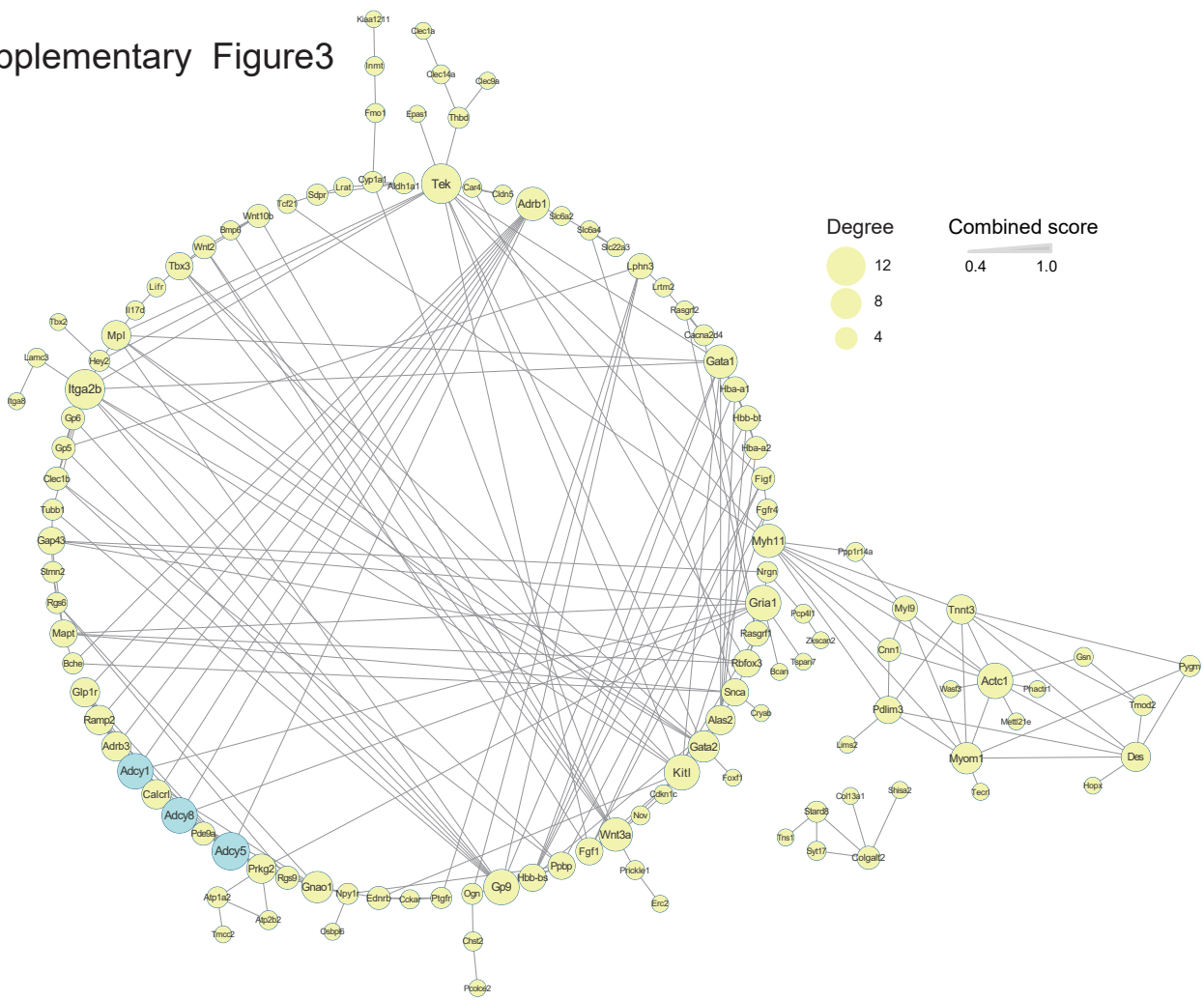

Supplement: Supplementary file 3 — Fig S3 [file JCMM-24-8248-s003.pdf]
