# Supplementary material for: Asiaticoside might attenuate bleomycin‐induced pulmonary fibrosis by activating cAMP and Rap1 signalling pathway assisted by A2AR
Source: J Cell Mol Med. 2020 Jun 16;24(14):8248–61. doi: 10.1111/jcmm.15505 (PMC7348182; doi:10.1111/jcmm.15505)

Supplementary Figure 4

A. Quality control for KO vs control group

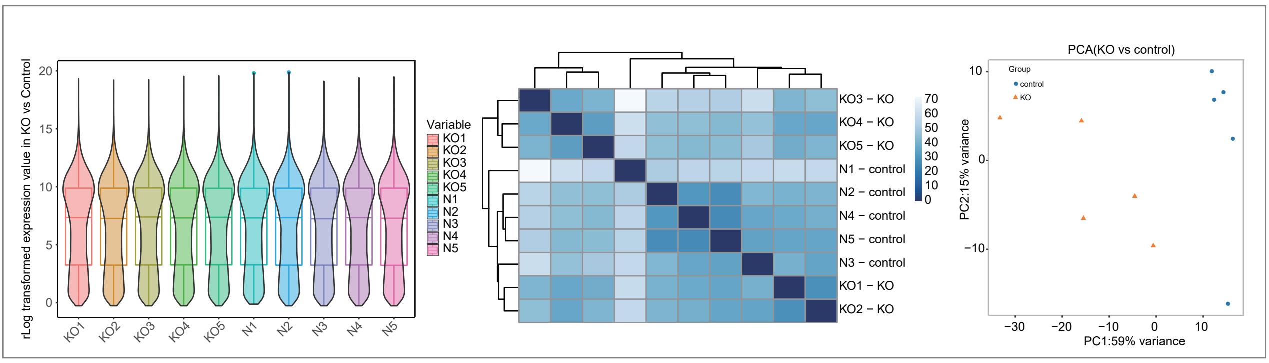

B. Quality control for KOB vs KO group

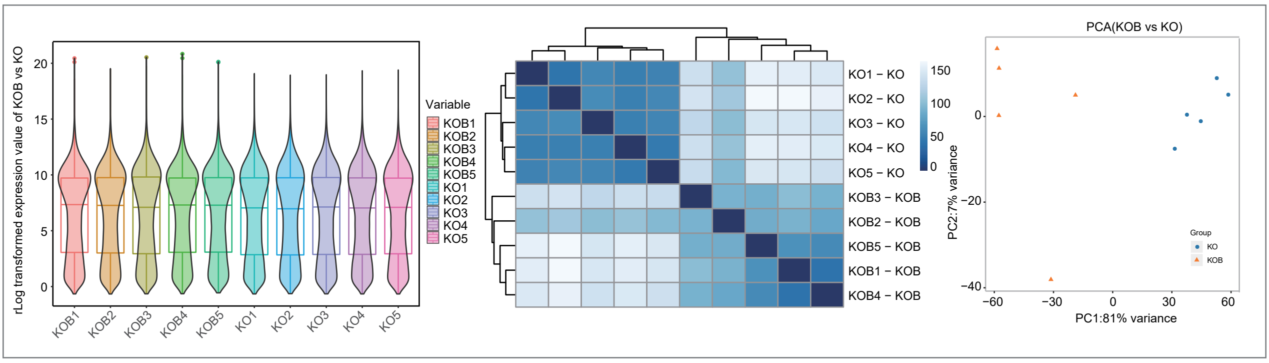

C. Quality control for KOAS vs KOB group

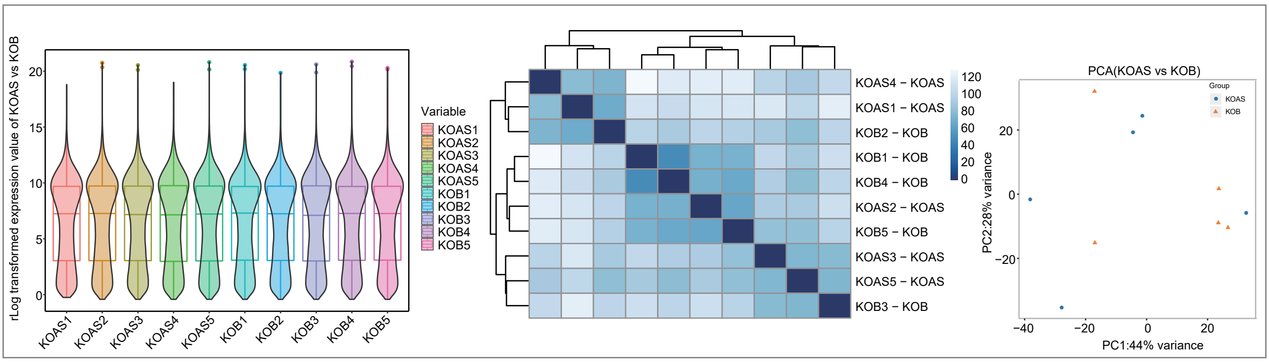

D. Quality control for KOAS vs AS group

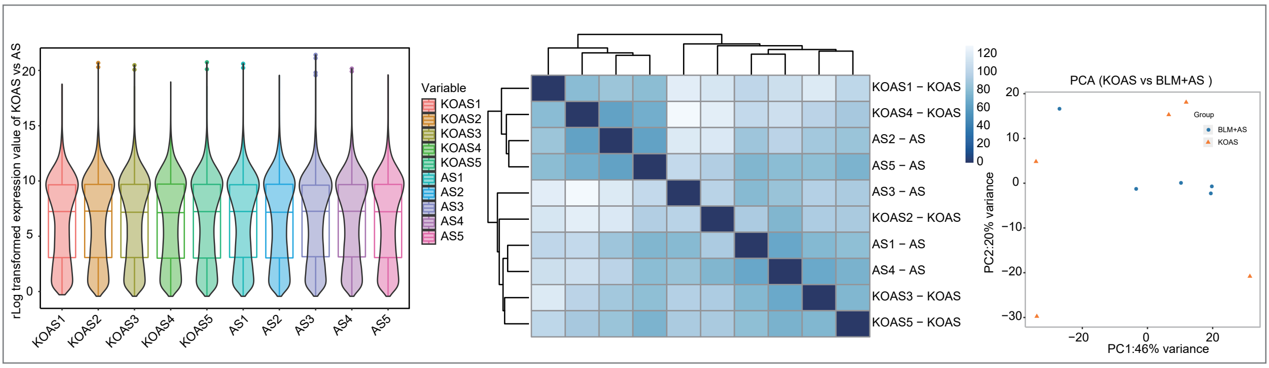

E. Quality control for KOB vs BLM group

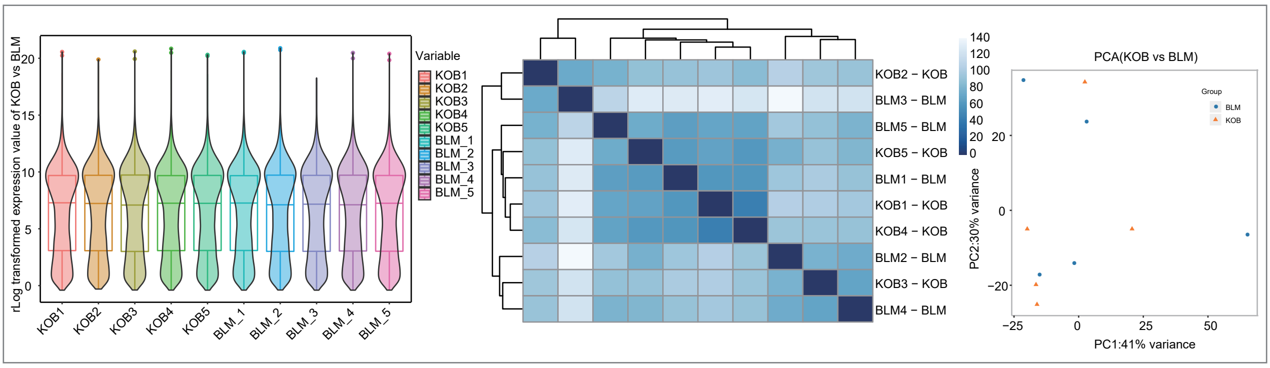

Supplement: Supplementary file 4 — Fig S4 [file JCMM-24-8248-s004.pdf]
