# Supplementary material for: Asiaticoside might attenuate bleomycin‐induced pulmonary fibrosis by activating cAMP and Rap1 signalling pathway assisted by A2AR
Source: J Cell Mol Med. 2020 Jun 16;24(14):8248–61. doi: 10.1111/jcmm.15505 (PMC7348182; doi:10.1111/jcmm.15505)
Supplement: Supplementary file 5 — Table S1‐S3 [file JCMM-24-8248-s005.docx]

**Supplementary Table1. DEGs of KOAS vs KOB group.**

| **Gene Symbol** | **log2FoldChange** | ***p*.adj** |
| --- | --- | --- |
| COL11A1 | 2.24 | 3.23E-07 |
| IGHV12-3 | 5.45 | 5.43E-06 |

| **Gene Symbol** | **log2FoldChange** | ***p*.adj** |
| --- | --- | --- |
| GM13443 | 4.72 | 8.28E-04 |
| AMD-PS4 | 2.69 | 2.10E-02 |
| UBTD1 | 2.38 | 2.10E-02 |
| GREM1 | 2.03 | 1.85E-02 |
| **ADCY1** | -4.88 | 1.24E-53 |
| **CAMP** | -3.52 | 4.82E-20 |
| GATA1 | -3.15 | 6.35E-04 |
| GM44891 | -3.05 | 2.97E-02 |
| GM37033 | -3.00 | 8.83E-03 |
| MT-ND4L | -2.91 | 2.61E-03 |
| GP9 | -2.85 | 8.28E-04 |
| TUBB1 | -2.53 | 5.07E-16 |
| GP5 | -2.43 | 6.35E-04 |
| MIR99AHG | -2.26 | 2.10E-02 |
| PPBP | -2.19 | 8.28E-04 |
| TREML1 | -1.81 | 3.48E-03 |
| GM26569 | -1.66 | 1.48E-02 |
| NRGN | -1.66 | 2.35E-02 |
| **RAP1** | -1.55 | 5.46E-15 |
| ITGA2B | -1.34 | 9.26E-04 |
| P2RX1 | -1.22 | 1.09E-02 |
| 4933406C10RIK | -1.05 | 8.83E-03 |
| DYNLT1C | -1.03 | 8.28E-04 |

**Supplementary Table2. DEGs of KOAS vs BLM+AS group.**

**Supplementary Table3. DEGs of KOB vs BLM group.**

| **Gene Symbol** | **log2FoldChange** | ***p*.adj** |
| --- | --- | --- |
| AMD-PS4 | -3.14 | 4.20E-02 |
| COL11A1 | -2.42 | 3.90E-02 |
| CTPS2 | -1.59 | 4.00E-02 |
| CYP26B1 | 1.50 | 4.20E-02 |
| DNAH8 | 1.65 | 5.98E-05 |
| ENTPD4B | 1.73 | 3.05E-04 |
| FBN2 | 1.74 | 4.20E-02 |
| GM13443 | 2.29 | 4.20E-02 |
| IGKV2-112 | 2.42 | 9.00E-03 |
| IGKV4-70 | 2.46 | 2.80E-02 |
| ISOC2B | 5.30 | 1.66E-04 |
| OAS1A | 5.61 | 2.30E-06 |
| OAS1G | 10.28 | 3.00E-03 |
| UBTD1 | 11.80 | 4.43E-04 |
